# Addition of Arsenic Trioxide into Induction Regimens Could Not Accelerate Recovery of Abnormality of Coagulation and Fibrinolysis in Patients with Acute Promyelocytic Leukemia

**DOI:** 10.1371/journal.pone.0147545

**Published:** 2016-01-26

**Authors:** Ye Zhang, SiJing Wu, Dan Luo, JianFeng Zhou, DengJu Li

**Affiliations:** Department of Hematology, Tongji Hospital, Tongji Medical College, Huazhong University of Science and Technology, Wuhan, Hubei, China; Queen's University Belfast, UNITED KINGDOM

## Abstract

**Aim:**

All-trans retinoic acid combined to anthracycline-based chemotherapy is the standard regimen of acute promyelocytic leukemia. The advent of arsenic trioxide has contributed to improve the anti-leukemic efficacy in acute promyelocytic leukemia. The objectives of the current study were to evaluate if dual induction by all-trans retinoic acid and arsenic trioxide could accelerate the recovery of abnormality of coagulation and fibrinolysis in patients with acute promyelocytic leukemia.

**Methods:**

Retrospective analysis was performed in 103 newly-diagnosed patients with acute promyelocytic leukemia. Hemostatic variables and the consumption of component blood were comparably analyzed among patients treated by different induction regimen with or without arsenic trioxide.

**Results:**

Compared to patients with other subtypes of de novo acute myeloid leukemia, patients with acute promyelocytic leukemia had lower platelet counts and fibrinogen levels, significantly prolonged prothrombin time and elevated D-dimers (P<0.001). Acute promyelocytic leukemia patients with high or intermediate risk prognostic stratification presented lower initial fibrinogen level than that of low-risk group (P<0.05). After induction treatment, abnormal coagulation and fibrinolysis of patients with acute promyelocytic leukemia was significantly improved before day 10. The recovery of abnormal hemostatic variables (platelet, prothrombin time, fibrinogen and D-dimer) was not significantly accelerated after adding arsenic trioxide in induction regimens; and the consumption of transfused component blood (platelet and plasma) did not dramatically change either. Acute promyelocytic leukemia patients with high or intermediate risk prognostic stratification had higher platelet transfusion demands than that of low-risk group (P<0.05).

**Conclusions:**

Unexpectedly, adding arsenic trioxide could not accelerate the recovery of abnormality of coagulation and fibrinolysis in acute promyelocytic leukemia patients who received all-trans retinoic acid combining chemotherapy.

## Introduction

Acute promyelocytic leukemia (APL) is a distinct subtype of acute myeloid leukemia (AML), and accounts for approximately 5–10% of cases of AML [1]. It is characterized by an excess of abnormal hypergranular promyelocytes in the bone marrow and other hematopoietic organs, and chromosomal translocation t(15;17)(q22;q21) leading to fusion of the genes encoding promyelocytic leukemia protein(PML) and retinoic acid receptor alpha (RARα) to generate the PML-RARα oncoprotein [2]. The abnormality of coagulation and fibrinolysis in APL is unique, and it account for early death in 10–30% of patients with APL [3].

The prompt combination of all-trans retinoic acid (ATRA) with chemotherapy has become a consensus regimen for treating newly diagnosed APL patients currently. The therapeutic efficacy of the regimen with ATRA and chemotherapy has been confirmed by a series prospective randomized clinical trials. The clinical complete remission rate was observed in 90%-95% of patients. 6-year disease-free survival rate is 68%, and 6-year overall survival rate is up to 83.9%[4–7]. Apparently, ATRA/chemotherapy combination regimen is superior to ATRA or chemotherapy alone. However, the death rate in early 28 days from diagnosis is still high, with hemorrhagic death at 5–11% being the major cause [8–10]. Since 1990s, the use of arsenic trioxide (ATO) has improved the clinical benefit of refractory or relapsed as well as newly diagnosed APL [11]. The regimens containing ATO was later approved by the US FDA for these refractory or relapsed APL. A randomized European Phase III trial compared a synergistic targeted therapy of ATRA plus ATO with ATRA plus standard chemotherapy. The results showed that the non-chemotherapy dual-differentiation agents for induction and consolidation therapy were superior to chemotherapy regimen in both two-year event-free and overall survival rates in patients with low-to-intermediate-risk APL [12]. Recently, another study by the Australasian Leukemia and Lymphoma Group using ATO superimposed on ATRA plus chemotherapy standard regimen for induction, while ATRA and ATO without chemotherapy for 2 cycle consolidation, also reported improved outcome with increased freedom from relapse and failure-free survival when compared to their previously reported ATRA/chemotherapy-based protocol. However, the rate of early death rate and overall survival were of no significant difference between the two groups [13].

Despite of the obviously improvement in survival rate after application of regimens with ATO, it is unclear whether or not these regimens can accelerate the recovery of abnormality of coagulation and fibrinolysis in patients with APL. The following retrospective study was trying to answer this question.

## Methods

### Patients

A total of 103 hospitalized patients with newly diagnosed de-novo APL were treated at Tongji hospital (Wuhan, China) during March 2008 to January 2015. These cases consisted of 60 males and 43 females, with age ranging from 14 to 74 years and a median of 37 years. 263 de novo AML (other than APL) were retrospectively analyzed at the same period as control group for comparisons of laboratory parameters at initial diagnosis. The diagnostic criteria of AML were based on the of World Health Organization Classification of Tumors- Pathology and Genetic of Tumors of Haematopoietic and Lymphoid Tissue (2008) and FAB (1976)[14]. Other inclusion criteria were: no serious liver disease or other hemorrhagic diseases, and no usage of anticoagulants during initial induction therapy. We collected the data from December 2014 to January 2015 and identify the information during and after data collection. This study has been approved by the ethics committee of Tongji Hospital Affiliated of Huazhong University of Science and Technology. Written informed consent was obtained from all enrolled subjects, including the next of kin on behalf of the minors recruited in our study.

ATRA 20 mg/m^2^ treatment was begun immediately at the time after APL was suspected. Chemotherapy and/or ATO (0.16 mg/kg/d, maximum≤10 mg/d) was prescribed according to prognostic risk stratification and individual physicians’ decisions. Therapeutic platelet or fresh frozen plasma (FFP) or cryoprecipitate transfusions were done only when clinically relevant bleeding occurred. Prophylactic platelet transfusion strategy was done when the platelet count was 30×10^9^/L or lower [15]. For platelet transfusions, patients only accepted random ABO-identical (non-HLA-typed) apheresis platelets when available. In China, one apheresis units are standardized to contain 2.5×10^11^ platelets or more with less than 5×10^8^ leucocytes. Prophylactic transfusion of FFP or cryoprecipitate mainly based on the fibrinogen level ≤ 1g/L. One unit cryoprecipitate was converted to 200ml plasma in favor of subsequent statistical analysis.

### Laboratory studies and clinical outcomes

The obtained information included case mix (age, gender,), clinical (initial bleeding events, early hemorrhagic death events, ATRA differentiation syndrome and consumption of transfused component blood), and laboratory variables [white blood cell (WBC) counts, platelet (PLT) counts, prothrombin time (PT), activated partial thromboplastin time (APTT), fibrinogen (Fbg), D-dimer, creatinine, uric acid, lactate dehydrogenase (LDH), bcr3 transcript type and blasts and promyelocytic percentage]. The rating criteria of bleeding were based on World Health Organization bleeding scale [16, 17]. Routine blood tests were carried out using a Sysmex XE-5000 Hematology Analyzer (Sysmex, Kobe, Japan) on EDTA-anticoagulated blood samples. The STA Compact Automated Hemostasis Analyzer (Diagnostica Stago, Gennevilliers, France) was used for detecting coagulation and fibrinolysis parameters, such as APTT, PT, Fbg level (Clauss method), D-dimer(Immuno-turbidimetric method). Blood biochemical test were done on COBAS INTEGRA 800 biochemical analyzer (Roche, Switzerland) on heparin-anticoagulated blood samples. Fusion gene transcript from chromosome aberrations was analyzed by reverse transcription polymerase chain reaction. Blasts and promyelocytic percentage was determined by microscopic examination of the bone marrow by two experienced physicians separately.

### Statistical analysis

According to induction regimens, APL patients were divided into two groups: ATO group (ATO+ ATRA +/no chemotherapy) and non-ATO group (ATRA +/no chemotherapy). Hemostatic variables and the consumption of transfused component blood were comparably analyzed between two groups using Mann-Whitney test for two-sample analysis.

Comparisons also were conducted between different prognostic risk groups of APL patients using Kruskal-Wallis test for multi-sample analysis. The prognostic risk stratification of APL is based on widely recognized risk evaluation standard which originate from the Italian GIMEMA and the Spanish PETHEMA trials: WBC≤10×10^9^/L and PLT>40×10^9^/L as low risk, WBC≤10×10^9^/L and PLT≤40×10^9^/L as intermediate-risk, and WBC≥10×10^9^/L as high-risk groups [18].

Hemostatic variables and the consumption amount of transfused component blood were expressed in median (range) format. All P-values were two-sided and less than 0.05 were considered as statistically significant. Statistical analysis was accomplished by SPSS software 20.0 (SPSS Inc., Chicago, IL, USA).

## Results

### Analysis of abnormality of coagulation and fibrinolysis

Compared to patients with other subtypes of de novo acute myeloid leukemia(AML), patients with APL had lower platelet counts and Fbg levels, significantly prolonged prothrombin time (PT) and elevated D-dimers(P < 0.001). The APTT median values of both groups were all in normal range (P > 0.05). Besides, APL patients had more bleeding events and early hemorrhagic death than AML patients (P < 0.001) (Table 1). Among the patients with APL, 48 patients bled at skin or soft tissue, 43 at oral or nasal, 11 at genitourinary system, 9 at central nervous system, 8 at pulmonary, 1 at gastrointestinal system and 1 at invasive sites.

**Table 1:** Evaluation of hemostatic parameters and Incidence of bleeding events in patients with APL or de novo AML (other than APL).

Parameters or events	APL	*de novo* AML	P value
	(n = 103)	(n = 263)	
**PLT [median (range)]**	24 (12–42)	31.5 (16–60)	<0.001
**PT [median (range)]**	16.8 (14.9–19.5)	14 (13–15.6)	<0.001
**APTT [median (range)]**	35.9 (28.9–40.5)	34.7 (29.3–39.6)	0.883
**Fbg [median (range)]**	1.41 (1.00–2.02)	3.81 (3.19–4.87)	<0.001
**D-Dimer [median (range)]**	19.9 (7.9–31.4)	0.8 (0.4–2.7)	<0.001
**initial bleeding grade I-II(n)**	70	91	<0.001
**initial bleeding grade III-IV(n)**	9	2	<0.001
**early hemorrhagic death(n)**	8	1	<0.001

APL, acute promyelocytic leukemia; AML, acute myeloid leukemia; PLT, platelet count, (40–100)*10^9^/L; PT, prothrombin time, 11.5–14.5 s; APTT, activated partial thromboplastin time, 28.5–41.5 s; Fbg, fibrinogen, 2.00–4.00 g/L; D-dimer, <0.5mg/ml; n, number of events.

Next, we analyzed the change in the trend of hemostatic variables in patients with APL. In order to minimize interference as much as possible, 14 cases were excluded due to early death (8 cases), pregnancy (2 cases), and withdrawing of treatment in 7 days after diagnosis (4 cases). The remaining cases (60 males and 43 females) were incorporated into the following research. The recording time points were respectively set at the first visit (day 0), and after treatment with ATRA/ATO/chemotherapy (day 1, day 4, day 7, day 10, day 13, day19 and day 25).

The median of PT returned to normal range after only 7 days of therapy. The median of Fbg kept rising step by step and had fallen in normal range since day 10. The elevated D-dimers showed a relatively slow downtrend and still maintained at a slightly high level in the fourth week of induction therapy (Table 2).

**Table 2:** Change in the trend of hemostatic parameters in patients with APL.

Time	PLT	PT	Fbg	APTT	D-dimer
	median (range)	median (range)	median (range)	median (range)	median (range)
**d0**	25 (13–40)	16.7 (14.8–19.8)	1.41 (1.01–2.06)	34.4 (28.7–40.2)	19.9 (8–33.1)
**d4**	42 (29–51)*	14.7 (13–15.9)*	1.88 (1.55–2.59)*	32 (27.6–36.2)*	6.5 (2.9–13.7)*
**d7**	37 (26–52)*	14.4 (12.6–15.1)*	1.89 (1.55–2.96)*	31.8 (27.2–36.7)	3.4 (1.4–8.3)*
**d10**	31 (20–48)	13.9 (12.6–14.8)*	2.41 (1.76–3.11)*	33.3 (27.5–38.3)	3.3 (1.7–7.2)*
**d13**	26 (18–44)	14.1 (12.6–14.8)*	2.9 (2.13–4.06)*	33.4 (29.5–37.9)	2.3 (0.8–4.8)*
**W3**	29 (17–45)	13.5 (11.9–14.4)*	3.21 (2–4.32)*	33.6 (28.941.4)	1.9 (0.6–2.9)*
**W4**	45 (31–104)*	13.5 (12.4–14.6)*	2.83 (2.11–4.42)*	34.7 (27.7–44.1)	1 (0.6–1.9)*

*, compare to the initial level, Wilcoxon signed rank test’s P value<0.01.

Based on whether to use ATO during initial treatment, all patients were divided into two groups: ATO group and non-ATO group. Patients of two groups had same baseline levels of fibrinolytic and hemostatic variables. There was no difference in change in the trend of D-dimer and PLT counts from day 0 to day 29. APTT median value of two groups kept in normal range all the time. Fbg level difference of two groups was lower from day 0 to day 10 and recovered to the normal level after day 13. The difference of Fbg level between two groups was not evident from day 0 to day 10, and then became obvious after day 13. But Fbg of two groups had recovered to the normal level at that time. The differences of PT median value of two groups were at day4 and day7. ATO group had a slightly higher level than that of non-ATO group (P<0.01). These results suggested that the application of ATO could not seemingly accelerate to correct abnormality of coagulation and fibrinolysis in APL patients (Table 3). Besides, no significant difference between the above two groups were found in the following clinical factors: mean value of blasts (2.86±2.84 vs. 1.95±2.70, P = 0.126), mean value of promyelocytic cells (82.87±11.15 vs. 84.48±13.99, P = 0.565), incidence of bcr3 transcript type (3/36 vs. 9/53, P = 0.347), incidence of elevated creatinine (5/34 vs. 8/51, P = 1.000), incidence of elevated uric acid (10/34 vs. 14/51, P = 1.000), distribution of low/ intermediate/ high-risk patients (9/18/9 vs. 10/25/18, P = 1.000) and incidence of ATRA differentiation syndrome (4/36 vs. 5/53, P = 1.000).

**Table 3:** Compared analysis of hemostatic parameters between non-ATO group and ATO group.

		Non-ATO group		ATO group		P value
		n	median (range)	n	median (range)	
**PLT**	d0	36	27 (15.3–43)	52	23 (10.7–34.5)	0.243
	d4	36	37.5 (29–51.5)	52	44 (29.4–51)	0.662
	d7	36	38 (21.3–52.5)	52	35.5 (29.5–53.3)	0.389
	d10	29	29 (24.5–46.5)	44	31.5 (18.7–49.2)	0.857
	d13	29	22 (15.5–38.5)	44	31.2 (19.5–51)	0.159
	W3	20	26 (15.5–44)	43	31 (17–46)	0.701
	W4	11	37 (28–52)	30	51.5 (32–126.3)	0.215
**PT**	d0	36	16.8 (4.4–20.6)	52	16.6 (15–19.2)	0.769
	d4	36	13.8 (11.7–14.9)	52	15.1 (14.1–16)	0.003
	d7	36	12.9 (11.2–14.6)	52	14.7 (14–15.4)	<0.001
	d10	29	13.2 (12.1–14.6)	44	14.1 (12.8–14.9)	0.082
	d13	29	14 (12.3–14.7)	44	14.3 (13.4–15)	0.144
	W3	20	13.4 (11.4–14.3)	43	13.6 (12.4–14.4)	0.434
	W4	11	13.5 (11.5–14.4)	30	13.6 (12.6–14.8)	0.717
**Fbg**	d0	36	1.55 (1.06–2.23)	52	1.37 (0.79–1.94)	0.116
	d4	36	1.88 (1.56–2.76)	52	1.88 (1.5–2.55)	0.799
	d7	36	1.89 (1.43–3.02)	52	1.93 (1.63–2.95)	0.725
	d10	29	2.56 (1.75–3.31)	44	2.39 (1.76–3.12)	0.774
	d13	29	3.95 (2.76–4.81)	44	2.52 (2.03–3.38)	0.002
	W3	20	3.99 (3.02–4.69)	43	2.88 (1.87–3.72)	0.009
	W4	11	3.9 (2.81–5.32)	30	2.71 (1.88–3.4)	0.023
**APTT**	d0	36	31.7 (27.7–39.9)	52	35.9 (29.7–40.2)	0.281
	d4	36	29.1 (25.3–33.5)	52	34.1 (29.9–37.1)	0.001
	d7	36	27.8 (25.7–33)	52	34.9 (29.3–41)	<0.001
	d10	29	31.5 (25.2–35.1)	44	34.5 (29.7–39.7)	0.010
	d13	29	32.8 (26.2–36.2)	44	33.6 (30.5–39.1)	0.083
	W3	20	31.3 (27.6–40.3)	43	34.6 (29–41.4)	0.447
	W4	11	30.8 (24.5–41.9)	30	34.9 (28.1–46.4)	0.632
**D-dimer**	d0	36	22.1 (7.2–39.6)	52	19.9 (8.4–30.7)	0.767
	d4	21	6.4 (2.8–14.5)	22	8.2 (2.7–12.8)	0.950
	d7	21	3.1 (2.3–7.7)	22	3.7 (1.1–9.1)	0.875
	d10	16	3.5 (1.8–6.8.)	18	3.2 (1.6–8.4)	0.959
	d13	16	2.4 (1.2–4.7)	18	1.9 (0.6–4.9)	0.567
	W3	7	2.3 (0.6–2.9)	16	1.8 (0.6–3.5)	0.922
	W4	4	0.8 (0.6–1.7))	12	1.2 (0.6–2.7)	0.684

The fibrinolytic or hemostatic variables of APL patients with different prognostic stratification were comparatively analyzed, too. The statistical differences existed in fibrinogen level at the time of initial diagnosis (day 0), PT at day 4, D-dimer at day 7. High risk group had a lower Fbg level at day 0 (P = 0.012) and longer PT at day 7 than low risk and intermediate risk groups. The value of PT, APTT and D-dimers at day 0 had no obvious difference among different risks groups (P>0.05). The whole change in the trend of fibrinolytic or hemostatic variables is approximately consistent during the induction treatment (Table 4). The result showed that the pace of coagulation recovery was not affected by prognostic stratification of APL.

**Table 4:** Change in the trend of hemostatic parameters in APL patients with different prognostic stratification.

		Low-risk		Intermediate risk		High-risk		P value
	Day	n	median (range)	n	median (range)	n	median (range)	
**PT**	d0	19	16 (13.3–19.9)	42	17.1 (14.4–19.7)	27	16.7 (14.9–20.8)	0.651
	d4	19	13.6 (11.7–15)	42	14.7 (13–16)	27	15 (14.2–16)	0.042
	d7	19	13.1 (11.2–15)	42	14.4 (12.9–15.2)	27	14.6 (13.2–15.1)	0.294
	d10	16	13.1 (12.3–14.6)	33	14 (12.7–14.9)	24	14.1 (12.6–14.8)	0.500
	d13	16	13.7 (12.4–14.6)	33	14.1 (13.1–14.9)	24	14.3 (13.4–14.9)	0.335
**Fbg**	d0	19	2.10 (1.35–2.97)	42	1.38 (1.02–1.94)	27	1.13 (I0.75–1.62)	0.012
	d4	19	2.06 (1.7–2.62)	42	1.82 (1.33–2.49)	27	2.01 (1.56–2.68)	0.426
	d7	19	2.25 (1.75–3.04)	42	1.89 (1.41–2.85)	27	1.85 (1.59–3.03)	0.379
	d10	16	3.19 (1.79–4.72)	33	2.31 (1.52–2.89)	24	2.42 (1.85–3.01)	0.144
	d13	16	3.44 (2.24–5.56)	33	2.45 (1.68–3.88)	24	2.91 (2.36–4.32)	0.102
**APTT**	d0	19	35.9 (29.7–40.7)	42	32.2 (27.5–40.7)	27	35.2 (30.1–39.4)	0.592
	d4	19	30.7 (28.8–34.2)	42	33.2 (27.7–38.5)	27	31.1 (26.8–36.3)	0.449
	d7	19	30.2 (26.9–35.8)	42	23.9 (27.5–38.8)	27	31.1 (26.6–36.6)	0.664
	d10	16	32.2 (27.9–38)	33	33.8 (26.6–37.8)	24	31.9 (27.7–38.4)	0.890
	d13	16	33.7 (28.4–38.1)	33	33.7 (30–38.6)	24	33.1 (29.5–37.1)	0.892
**D-dimer**	d0	15	18.9 (9.7–39.6)	38	23 (7.8–35.6)	20	17.3 (7.9–28.1)	0.623
	d4	7	1.6 (0.5–5.1)	21	9.2 (4.5–15.7))	15	7 (2.9–13.9)	0.067
	d7	7	2.2 (0.5–7.3)	21	6.5 (2.9–12.8)	15	2.5 (1–6.2)	0.047
	d10	7	1.6 (1.2–3.2)	17	5.8 (2.1–16.7)	10	3.5 (2.4–4.7)	0.063
	d13	7	1.5 (0.6–2.8)	17	2.4 (0.9–8.4)	10	2.4 (0.6–4.2)	0.454

### Analysis of consumption of transfused component blood

The median transfusion volume of plasma were 1200 ml/week (interquartile range 150–2350 ml/week) in the first week, but decreased sharply from the second week. In contrast, the median transfusion amount of platelet maintained at 1.5 unit/week during the first three weeks of induction therapy, and finally declined in the fourth week (Table 5).

**Table 5:** Compared analysis of consumption of component blood between non-ATO group and ATO group.

Component	Time	All	Non-ATO group		ATO group		P value
	Week	median (range)	n	median (range)	n	median (range)	
**PLT**	W1	2.0 (1.0–3.0)	12	0.5 (0–2.5)	45	2.0 (1.0–3.0)	0.297
**(unit)**	W2	2.0 (1.0–2.0)	12	2.0 (1.0–2.0)	44	1.5 (1.0–2.0)	0.818
	W3	1.0 (1.0–2.0)	12	2.0 (0.75–2.0)	44	1.0 (1.0–2.0)	0.325
	W4	0 (0–1.0)	9	0 (0–1.0)	37	0 (0–1.0)	0.711
**Plasma**	W1	1200 (150–2350)	12	2200 (0–2875)	45	900 (0–2175)	0.355
**(ml)**	W2	0 (0–150)	12	0 (0–212.5)	44	0 (0–0)	0.98
	W3	0 (0–0)	12	0 (0–0)	44	0 (0–0)	0.637
	W4	0 (0–0)	9	0 (0–0)	37	0 (0–0)	0.830

There were slightly differences between non-ATO group and ATO group in the consumption of PLT and plasma transfusion. But no statistical significance was found in two groups (Table 5). The result indirectly shows that the most serious abnormality of coagulation appears in the early stages of the treatment. The pace of improvement was not related with the application of ATO in induction regiments.

The consumption of PLT and plasma transfusion was higher in high-risk group and intermediate-risk group than low-risk group in the first week. But statistical differences only were found in the platelet transfusion (P<0.05) (Table 6).

**Table 6:** Change in the trend of consumption of component blood in patients with APL with different prognostic stratification.

Component	Time	Low-risk		Intermediate risk		High-risk		P value
	Week	n	median (range)	n	median (range)	n	median (range)	
**PLT**	W1	11	0.5 (0–1.25)	27	2.0 (1.0–3.0)	19	2.0 (2.0–3.0)	0.005
**(unit)**	W2	11	1.0 (0–2.0)	27	2.0 (1.0–2.0)	18	2.0 (1.0–2.0)	0.438
	W3	11	1.5 (0–2.25)	27	1.0 (1.0–2.0)	18	2.0(1.0–2.0)	0.842
	W4	10	0 (0–1.0)	21	0 (0–1.0)	15	0 (0–1.0)	0.747
**Plasma**	W1	11	0 (0–2712.5)	27	900 (0–2500)	19	1200 (775–2025)	0.501
**(ml)**	W2	11	0 (0–100)	27	0 (0–150)	18	0 (0–75)	0.955
	W3	11	0 (0–0)	27	0 (0–0)	18	0 (0–0)	0.914
	W4	10	0 (0–0)	21	0 (0–0)	15	0 (0–0)	0.368

## Discussion

APL-associated coagulopathy is more complex than simple disseminated intravascular coagulation (DIC). Activation of the clotting system, increased fibrinolytic activity and non-specific protease activity, with hyperfibrinolysis predominating are all included [19]. Recent studies have revealed that the unique abnormalities of coagulation and fibrinolytic function are associated with increased amounts of tissue factor(TF), cancer procoagulant (CP) as well as elements of the fibrinolytic system, including tissue plasminogen activator, annexin A2, and plasminogen activator inhibitor type 1 expressed by leukemic promyelocytes in APL [20].

In our study, abnormalities of the 103 newly diagnosed patients with APL in routine hemostatic variables include low platelet counts, prolonged PT, low Fbg levels, elevated D-dimers, more bleeding events and higher early hemorrhagic death rate, which are consistent with previous reports [19, 21, 22]. Low platelets are usually due to a result of impaired platelet production and consumption. Increase in D-dimer and decrease in the fibrinogen level are the evidence of hyperfibrinolysis [20, 23]. Clearly, bleeding events and high early hemorrhagic death rate are resulted from the coagulopathy described above.

Several studies have already confirmed the change in the trend of hemostatic variables during the first or second weeks of treatment [24, 25]. Our study provides other convincing evidences, such as significant recovery of abnormal hemostatic markers and phasedown of consumption of transfused component blood, which supported the restoration of coagulation and fibryolysis by induction therapy.

Recent studies revealed that ATRA and ATO could specially bind to RARα and PML moieties of PML-RARα oncoprotein respectively and leading to their degradation [26, 27]. Furthermore, double induction of ATRA and ATO could cause APL cells differentiation, apoptosis [28–30]. Several studies have confirmed benefits of ATRA-ATO combination for newly diagnosed APL in long-term follow-up [12, 13, 29, 31]. Compared to those treated with either single agent, more encouraging outcomes were achieved in pilot studies with patients receiving dual induction of ATRA and ATO, including shorter time needed to achieve CR and higher rate of CR, enhanced 5-year disease-free survival, event-free survival rates and overall survival rate, less hematologic toxicity and fewer infections but more hepatic toxicity [12, 32–34]. Another study also showed improved clearance of PML-RARA transcripts in patients receiving the combination therapy of ATRA and ATO [29]. Most studies focused on the therapeutic effect comparison of patients with APL by different induction regiments including ATRA and/or ATO. Our study paid more attention to the change in the trend of coagulation and fibrinolysis during the initial treatment. We found that adding ATO into induction regiments neither accelerate the recovery of abnormality of coagulation and fibrinolysis nor decrease the consumption of transfused component blood in patients with APL.

Previous reports suggested high WBC count (>10×10^9^/L) as an adverse prognostic factors for bleeding complications in APL [35, 36]. However, our results did not find that the pace of coagulation recovery was affected by prognostic stratification of APL. But we found that the demand amounts of PLT and plasma transfusion increased in high-risk group and intermediate risk group than low-risk group in the first week.

The limitations of this study were the relatively small sample size, missing data on some patients, and biases of judgment on bleeding diathesis which will directly affect the transfusion demand of PLT and plasma. More APL specific and sensitive laboratory tests such as, levels of thrombin antithrombin complex, prothrombin fragment, amount of tissue factor and cancer procoagulant and plasminogen activator and annexin A2 levels were not included this time due to the limitation of retrospective study, and we will make up in the follow up future studies.

In conclusion, ATO/ATRA plus chemotherapy regimen relieves the coagulopathy burden in the induction period. Our study found unexpectedly that adding ATO could not accelerate the recovery of abnormality of coagulation and fibrinolysis in APL patients. Moreover, it was necessary to pay more attention to satisfy the high demand of component blood transfusion in initial treatment, which will substantially decrease the serious bleeding episodes related to abnormality of coagulation and fibrinolysis.
